# Gomphosis, a Barrier That Cannot Be Swept Away

**Published:** 1900-02

**Authors:** B. H. Catching

**Affiliations:** Atlanta, Ga.


### Abstracts and Translations.

### GOMPHOSIS, A BARRIER THAT CANNOT BE SWEPT AWAY.[1]

BY B. H. CATCHING, D.D.S., ATLANTA, GA.

It is not the purpose of this paper to throw the least obstacle in the way of dental progress, but to call attention to a barrier to professional progress.

Those who are striving for advancement are so few, comparatively, that the combined efforts are needed to uphold a cause to which the large majority of its followers are indifferent, or are real hinderances.

[1] This paper having been published abroad, it is given here at the request of Dr. Catching, made a short time before his death.—EDITOR.

It may be so with all professions, but it does seem that dentistry has a larger proportion of inactive members than any other.

Is there a reason for this beyond mere indifference? I think there is, but they are not cognizant of it as such. It is a barrier that stands in the way of those who would see that dentistry is placed along with other specialties. It is of this barrier that I wish to speak, and, in so doing, I am not prepared to suggest a remedy, for there is none. It was placed by Infinite law, and the finite cannot change it.

It is an empty sound to hear from all sides that dentistry has made the most wonderful strides of any profession, and that it now ranks with the most learned. The progress, dentally speaking, has been limited, save in a mechanical way. Orally speaking, however, the progress has been rather wonderful.

Proud of this as we are, and as elevating as it is, yet we must revert to dentistry proper, which is the field of the rank and file, to find the barrier to higher attainments; a barrier that will ever exist to prevent the advancement of the dentist to the position of the oculist and aurist.

With the oral surgeon there is no limit or bound to his advancement; there is no barrier. But to the dentist it is, "so far shalt thou go, and no farther."

Having our bacteriologists, histologists, and microscopists is well, and to them much praise is due; through them it will be that true dentistry, *preventive dentistry*, will come in time. But they, with the institutions of learning and with literature, cannot remove the obstruction and rank dentistry with other specialties. They give position before the learned. There is a broad field of usefulness for the dentist, standing, as he does, at the threshold of health; and he covers this field well; nevertheless, he is circumscribed.

With the masses, and to a degree with the classes, gomphosis will hold him to a sphere from which he cannot rise to that position of prominence which is the desire of all who aspire to things higher, better, and nobler.

The classes cannot be turned away from this peculiar anatomical relation. They cleave to it as a panacea for all dental ills, and not at all as a last resort, but simply as the only means for relief. They care not for professional scientific attainments; they care not for the loss of the dental organs; they feel that they are not maimed for life; they are satisfied with the thought of being relieved from suffering, having in mind all the while the dental substitute.

The classes are tolerant, a few will seek the most skilful; but they will not often go to the limit of the operator's ability to save, preferring to endure for a period rather than suffer for a season.

Must I say it? Is it not because of gomphosis that the dentist rests with the idea that he has a last resort, a mechanical one, and does not strive for higher attainments, feeling that the incentive to broader culture does not exist; satisfied with a knowledge of dental technics, coupled with some information on pathology and therapeutics, and that back of all is a bridge or plate? Verily, this peculiar relation of the teeth stands as a barrier to his progress beyond mechanics.

How different it would be if the anatomical relation of the teeth to the bone were different, so different that they could not be removed without a surgical operation of a major degree, or, if removed, they could not be substituted.

The real value of a tooth, by the majority, is in proportion to the pain of extraction. Anæsthetics have aided much in reducing the value. With a certain class the value is rated by the appearance; with another class, and the only true class, but a small one, the value is rated by the true service of the tooth.

With all classes the loss of a tooth is not looked upon as a calamity that cannot be rectified. This indifference fixes the standard of the dentist below that of other specialists.

For comparison, take the sense of hearing. What a calamity it is to lose this sense even in one ear. Every effort and all means are used to save it. The limit of the purse is gone in seeking skill, if necessary, for there can be no su stitute; the only thing is to save, if possible.

Suppose an artificial eye could be substituted for a natural one with the same degree of functional activity that an artificial tooth possesses, would it not lower the profession of the oculist to a standard far below its present exalted position? Would it not invite hordes into the vocation whose only claim would be that of making eyes? Would there be sufficient incentive for the oculist to strive for higher attainments surgically? Would it not be, "I will try, and if I fail you can have a substitute"? So it is with the dentist; circumscribed without the proper incentive; thrown, as a last resort, upon a mechanical substitute.

Verily, gomphosis is a barrier to professional progress that cannot be swept away.

